# The Impact of Human Papilloma Viruses, Matrix Metallo-Proteinases and HIV Protease Inhibitors on the Onset and Progression of Uterine Cervix Epithelial Tumors: A Review of Preclinical and Clinical Studies

**DOI:** 10.3390/ijms19051418

**Published:** 2018-05-09

**Authors:** Giovanni Barillari, Paolo Monini, Cecilia Sgadari, Barbara Ensoli

**Affiliations:** 1 Department of Clinical Sciences and Translational Medicine, University of Rome Tor Vergata, 1 via Montpellier, 00133 Rome, Italy; 2 National HIV/AIDS Research Center, Istituto Superiore di Sanità, 299 viale Regina Elena, 00161 Rome, Italy; paolo.monini@iss.it (P.M.); cecilia.sgadari@iss.it (C.S.); barbara.ensoli@iss.it (B.E.)

**Keywords:** HPV, uterine CIN, uterine cervical carcinoma, MMP, HIV-PI

## Abstract

Infection of uterine cervix epithelial cells by the Human Papilloma Viruses (HPV) is associated with the development of dysplastic/hyperplastic lesions, termed cervical intraepithelial neoplasia (CIN). CIN lesions may regress, persist or progress to invasive cervical carcinoma (CC), a leading cause of death worldwide. CIN is particularly frequent and aggressive in women infected by both HPV and the Human Immunodeficiency Virus (HIV), as compared to the general female population. In these individuals, however, therapeutic regimens employing HIV protease inhibitors (HIV-PI) have reduced CIN incidence and/or clinical progression, shedding light on the mechanism(s) of its development. This article reviews published work concerning: (i) the role of HPV proteins (including HPV-E5, E6 and E7) and of matrix-metalloproteinases (MMPs) in CIN evolution into invasive CC; and (ii) the effect of HIV-PI on events leading to CIN progression such as basement membrane and extracellular matrix invasion by HPV-positive CIN cells and the formation of new blood vessels. Results from the reviewed literature indicate that CIN clinical progression can be monitored by evaluating the expression of MMPs and HPV proteins and they suggest the use of HIV-PI or their derivatives for the block of CIN evolution into CC in both HIV-infected and uninfected women.

## 1. Role of the HPV-E5, E6 and E7 Proteins in the Development of Uterine Cervical Pre-Cancer and Cancer Lesions

Infection by human papilloma virus (HPV) is frequent in sexually active women and plays a driving role in the development of proliferative/dysplastic or tumor lesions of the uterine cervix [1]. In particular, HPV is present in almost the totality of uterine cervical carcinoma (CC), which is the third most frequent malignancy in women worldwide [1].

The uterine cervix is composed by two different types of epithelium. Specifically, the endocervix is lined by a simple glandular epithelium, while the ectocervix is lined by a squamous epithelium constituted by superficial and deep (basal) layers [2]. At puberty, the endocervical glandular epithelium proximal to the ectocervix is replaced by the stratified squamous epithelium typical of the ectocervix. This trans-differentiated area is known as the “transformation zone” [2].

The proper stratification of uterine ectocervical epithelium and “transformation zone” is maintained through a tight control of epithelial cell growth, differentiation and locomotion. Specifically, epithelial cells originate in the deep layers, maturate and migrate to the superficial layer where they fully differentiate and desquamate in a continuous, self-renewing process [2]. 

This dynamic equilibrium is compromised by HPV which, in the presence of cervical micro-wounds, can access the deep layers and enter immature, proliferating cells [3,4]. There, the circular, double-stranded HPV DNA is maintained as an extra-chromosomal element (episome) which replicates via the synthetic machinery of host cell genome [3,4]. As such, HPV forces infected immature cervical cells to remain in a proliferative state and impedes their terminal differentiation. In particular, during HPV DNA replication, the HPV early genes, including those coding for the E5, E6 or E7 proteins, are transcribed [4]. Although HPV-E1 and E2 proteins support viral replication in the basal cells at a low copy number [5], HPV-E5, E6 or E7 are produced at levels sufficient to functionally impair host cell growth-regulatory or differentiation factors [4,6].

In particular, the HPV-E5 protein interferes with the signaling pathways of epidermal growth factor (EGF), a powerful inducer of epithelial cell survival, growth and locomotion [6]. Specifically, E5 promotes the sustained activation of EGF receptor, resulting in the triggering of protein kinases including the serine/threonine kinase AKT which, in turn, leads to epithelial cell survival and proliferation [6]. In this context, HPV-E5 down-regulates the expression of the p21 or p27 cell cycle inhibitors and counteracts cell death induced by the Tumor Necrosis Factor-Related Apoptosis-Inducing Ligand (TRAIL) or Fas-Fas ligand signaling [6]. Noteworthy, the amplification of EGF signaling promoted by HPV-E5 also inhibits epithelial cell differentiation via a reduction of keratinocyte growth factor expression [7].

**Figure 1:**
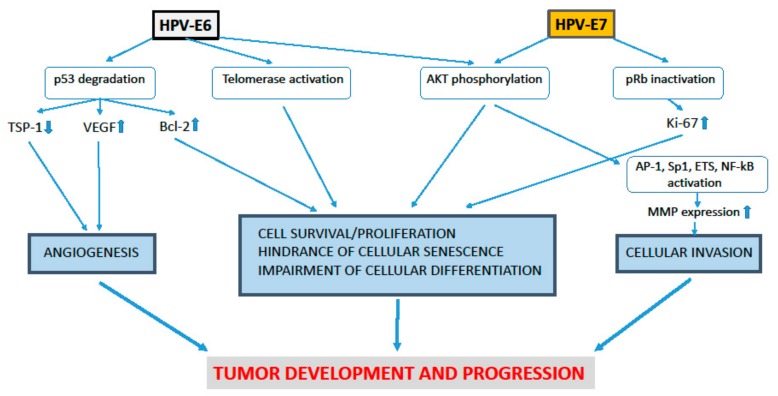
Tumorigenic effects of the E6 or E7 proteins of high-risk human papilloma viruses (HR-HPV). Arrows symbolize directions of connections. The E6 protein promotes p53 degradation, and this is followed by the down-regulation of thrombospondin (TSP)-1, or the up-regulation of vascular endothelial cell growth factor (VEGF) which, in turn, lead to angiogenesis. At the same time, E7 inactivates the retinoblastoma protein (pRb), and this increases Ki-67 protein levels. Noteworthy, both E6 and E7 induce AKT phosphorylation. This, together with the activation of telomerase or the up-regulation of Bcl-2 promoted by E6, and the increase of Ki-67 induced by E7, causes cell survival and proliferation. Moreover, the phosphorylation of AKT triggered by either E6 or E7 activates transcription factors including Activator Protein (AP)-1, Sp (Specificity protein)-1, ETS or Nuclear Factor-kappa B (NF-κB) which, in turn, induce matrix-metalloproteinase (MMP) expression and cellular invasion. Cell survival/ proliferation/ invasion and angiogenesis promoted by the E6 or E7 protein of HR-PV lead to tumor development and progression.

Concerning HPV-E7, it binds and inactivates the retinoblastoma tumor suppressor protein (pRb), hence preventing infected cells from exiting the cell cycle and differentiating (Figure 1) [8]. 

At the same time, HPV-E6 directs the host cell tumor suppressor protein p53 toward degradation through the cellular proteasome (Figure 1) [9]. In doing so, E6 increases the intracellular levels of the anti-apoptosis Bcl-2 protein which is normally repressed by p53 (Figure 1) [9]. In addition, E6 triggers the activity of telomerase, an enzyme that prevents replicative senescence by stabilizing the length of the chromosomes’ end (Figure 1) [9].

Moreover, the HPV-E5, E6 or E7 proteins stimulate the cyclooxygenase (COX)-2 inflammatory pathway which, as for EGF, counteracts the apoptosis of epithelial cells and promotes their proliferation [6]. 

Altogether, these activities of E5, E6 or E7 render HPV-infected cells insensitive to the regulatory signals of normal growth and block apoptosis otherwise occurring due to the sustained, uncontrolled cellular proliferation [6,8,9].

In about 80% of the cases, HPV-induced cell proliferation remains subclinical, augmenting cervical epithelium thickness or causing benign flat warts [10]. In the other cases, HPV-E5, E6 and E7 accelerate the growth rate of immature cervical basal cells and their migration to the superficial layer. This leads to the development of squamous intraepithelial lesions (SIL) [2,11].

According to the Bethesda system for reporting cervical cytological diagnosis, there are two kinds of SIL: low grade-SIL (L-SIL), which is the histologic correlate of productive HPV infection and high grade-SIL (H-SIL), that represents the early stages of HPV-induced carcinogenesis [2,11].

L-SIL corresponds histologically to grade 1 cervical intraepithelial neoplasia (CIN1), a mild dysplasia characterized by proliferating immature basal cells constituting 1/3 of the cervical epithelium. Because of HPV replication, superficial cells display koilocytosis (cave-like vacuoles around enlarged, hyper-chromatic nuclei) and altered keratinization [2,11].

H-SIL comprises grade 2 and 3 CIN (CIN2 and CIN3). The former is a moderate dysplasia in which basal cell types represent the 2/3 of cervical epithelium and koilocytosis and dyskeratosis are more evident than in CIN1; whereas, CIN3 is a severe dysplasia, characterized by proliferating basal-like cells having abnormal nuclei and atypical mitoses, which occupy the 2/3 of the entire epithelium, including the superficial layers [2,11].

Generally, an effective immune response to HPV develops within months after infection, resulting in viral clearance [3,10]. Thereafter, p53 and pRb function in the basal layers is restored and epithelial cell growth and differentiation return to normality. This generally occurs with low-risk HPV types, such as HPV6 or HPV11, whose DNA remains episomal [10].

In contrast, persistent infection with high risk (HR)-HPV, including HPV16 and HPV18, can be followed by the integration of viral DNA into the host cell genome [5]. The probability of having persistent HR-HPV infection augments progressively with higher viral load [12] and it is favored by E5, E6 or E7 activities permitting HR-HPV escape from host immune surveillance. In particular, both the E5 and E7 proteins of HR-HPV can impair major histocompatibility complex (MHC)-restricted presentation of viral peptides to cytotoxic or helper T lymphocytes [13,14]. Specifically, E7 down-regulates the expression of MHC class I molecules, while E5 arrests them in the Golgi apparatus, reduces their transport to the cell surface and/or diminishes MHC class II expression [13,14]. Moreover, either the E6 or E7 protein of HR-HPV inhibits the production of the immune-stimulatory, anti-viral interferons and reduces the synthesis of proteins attracting antigen-presenting macrophages, dendritic or Langerhans cells to the infected area [13]. In addition, HR-HPV-E5, E6 or E7 can trigger molecular pathways leading to the shift from cellular to humoral immune response, the silencing of inflammation, the impairment of natural killer cell or natural killer T cell activity and the recruitment of immune suppressive regulatory T cells [13,14].

HPV integration into cellular DNA causes the deletion of the *HPV-E2* gene and the consequent overexpression of *HPV-E6 or E7* [5]. As for *HPV-E2*, also the *E5* gene of HPV is often deleted upon the integration of HPV DNA in the host cell genome [6]. Thus, at variance with *E5*, the *E6* and *E7* genes are permanently expressed during HPV infection, being indispensable for the maintenance of the transformed cell phenotype. For this reason, E6 and E7 are considered as the main transforming proteins of HPV [7,8,9].

In fact, as a result of *HPV-E6* or *E7* overexpression caused by *E2* gene deletion, the disturbance of cervical epithelial cell maturation and stratification is exacerbated [5]. In this context, cellular key mitotic checkpoints are impaired, leading to genomic instability, accumulation of secondary mutations and aneuploidy in infected cells [15,16,17,18]. Subsequently, the entire cervical epithelium is replaced by poorly differentiated cells displaying abnormal nuclei and atypical mitoses [2,11]. Later on, some of these cells acquire a “spindle” morphology and degrade the epithelial basement membrane, giving rise to the onset of an invasive cancer, whose predominant histological type is squamous cell carcinoma [2,11].

Interestingly, CC develops mainly in uterine cervical “transformation zone”, which is rich in immature, highly proliferating and HPV-sensitive basal cells [2]. Noteworthy, as for other tumor settings [19], CIN evolution into a true malignancy is accompanied by the formation of new blood vessels (angiogenesis) at the stromal/epithelial junction of CIN lesions [20,21]. Specifically, endothelial cells lining the lumen of the pre-existing vessels invade the vascular basement membrane, sprout, proliferate and migrate in the extra-vascular space, where they organize into hollow cords permitting blood influx [20,21]. These newly formed vessels nourish the growing tumor and provide additional routes for CC metastasis [20,21]. Accordingly, higher intra-tumor vessel density is associated with CC aggressiveness or recurrence and poorer patient survival [22,23].

It is of note that HPV infection has an important role also in CC-associated neovascularization. In particular, following p53 degradation promoted by HPV-E6, p53-induced genes encoding for angiogenesis inhibitors, such as thrombospondin (TSP)-1, are no longer transcribed; whereas, the p53-repressed genes of angiogenic factors, including vascular endothelial growth factor (VEGF), are up-regulated (Figure 1) [21]. Of interest, also HPV-E5 can promote VEGF expression and this is due to E5 capability of triggering both EGF and COX-2 signaling [6].

However, it should be highlighted that HPV infection progresses to cancer only in a small percentage of cases and that CIN lesions can also stabilize or regress [24]. In particular, the natural history of CIN1 includes regression (60% of cases), persistence (30%) and progression to CIN3 (10%) [24]. The like-hood of CIN2 regression is 45%, persisting 30% and progressing to CIN3 or invasive CC are 20% and 5%, respectively [24]. Concerning CIN3, about 35% of cases regress, while 10–15% evolve into invasive CC [24].

The risk of CIN progression to invasive CC is increased by the use of oral contraceptives, smoking, early age at first sexual intercourse, multiple sexual partners, repeated parity and co-infections [25,26,27]. To this regard, women infected by both HR-HPV and the human immunodeficiency virus (HIV)-1 have a higher incidence of uterine CIN and CC, as compared to their HIV-negative counterparts [28,29,30,31,32,33,34,35]. In addition, HR-HPV/HIV-doubly infected women have lower regression rates from high-grade to low-grade CIN, or from low-grade CIN to normal epithelium [31] and faster progression from low-grade to high-grade CIN [28,35]. Consistently, the median age of HIV-positive CC patients is much lower than in HIV-negative CC patients [36]. Furthermore, CIN recurrence after treatment is particularly frequent in HR-HPV/HIV-doubly infected women [31]. Because of these findings, uterine CC is considered an Acquired Immune Deficiency Syndrome (AIDS)-defining disease [37].

Indeed, both the incidence and the progression rates of cervical lesions increase with the impairment of immune functions promoted by HIV, as indicated by the decrease in CD4^+^ T cell counts [28,35,38]. Certainly, the lack of an effective immune response to HR-HPV may favor its persistence, which is the main risk factor for CC development [1,38]. Nevertheless, HIV-1 is likely to have also a direct role in CIN progression to CC. In particular, results from in vitro studies indicate that the HIV-1 trans-activator (Tat) can up-regulate HR-HPV E6 or E7 expression, thereby decreasing the protein levels of cellular onco-suppressors and accelerating epithelial cell growth [39,40,41,42]. In addition, HIV-1 Tat may also favor the angiogenic switch of high-grade CIN, either because of its direct angiogenic effects [43] or, again, via the up-regulation of HR-HPV E6 or E7 expression [21]. 

Due to the dynamism of cervical lesions, cytological testing (PAP test) or histopathology is not always sufficient to assess the risk of CIN progression or its regression. Thus, morphological examination of cervical samples must be accompanied by the detection of risk biomarkers for high-grade CIN or CC, including the presence of HR-HPV DNA [44] and HR-HPV E6 and E7 overexpression [45,46]. Additional analyses are employed to monitor cellular (host) proteins whose synthesis in cervical lesions is de-regulated upon p53 and pRb functional inactivation. These include the cyclin-dependent kinase inhibitor p16INK4a and the proliferation antigen Ki-67 (Figure 1) [47,48,49]. In particular, the progression of CIN lesions can be predicted by the combination of low pRb/p53 and high Ki-67/p16INK4a expression in the basal layers of the cervical epithelium [47,48,49]. Recent data suggest evaluating the profile of specific cellular micro-RNAs that have an important role in cervical carcinogenesis and are modulated by the E5, E6 or E7 proteins of HR-HPV [50]. Further prognostic information is provided by CD4^+^ T cell counts in HPV/HIV doubly-infected women [35,36,38] and by an increase in the expression and/or activity of the cellular matrix-metalloproteinases (MMPs), which occurs in both HIV-positive and HIV-negative women.

## 2. The MMPs and Their Role in the Progression of Uterine Cervical Pre-Cancer and Cancer Lesions

MMPs are a family of peptidases defined by the presence of a zinc ion at the catalytic site and by the capability of degrading the protein components of the basement membrane and extracellular matrix (ECM) [51,52,53].

Human MMPs consist of distinct proteases which are classified in soluble and membrane-anchored [51,52,53].

Soluble MMPs contain an NH_2_-terminal secretion leader sequence and they include collagenases, stromelysins, matrilysins, the metalloelastase and gelatinases. Among the latter are MMP-2 and MMP-9, two powerful enzymes which can degrade a wide variety of basement membrane and ECM molecules [51,52,53].

The membrane-anchored MMPs (also called membrane type MMPs, MT-MMPs) display a COOH-terminal trans-membrane domain linked to a cytoplasmic tail and comprise 6 members [54]. The best known is MT1-MMP which, in addition to breaking down the ECM, is the main activator of MMP-2 [54].

Thus, ECM molecules are not the only target of MMPs, which can modify or digest many other extracellular or intracellular proteins, including enzymes, cytokines, chemokines and adhesion or growth factor receptors [52,55].

Because of these properties, MMPs are deeply involved in tissue remodeling or repair, in the regulation of cell growth, survival or differentiation and in reactive processes such as immunity and inflammation [56].

In physiological conditions, MMP expression is absent or very low in most tissues, being induced or up-regulated only during reactive/reparative processes [51,52,53,57]. In these events, growth factors, hormones or cytokines trigger signaling molecules that, in turn, activate transcription factors such as Activator Protein (AP)-1, ETS, Specificity protein (Sp)-1, and/or Nuclear Factor-kappa B (NF-κB) [58,59,60,61,62,63,64,65,66,67,68]. These activation pathways depend on the different cell types synthesizing MMPs and are influenced by cell-to-cell contacts and cell-ECM interactions [51,52,53].

In order to keep under control their potent activity, soluble MMPs are secreted as latent zymogens (proMMPs), which are converted into the active form around their producing cells, this providing a spatial control of MMP function [51,52].

Active soluble MMPs, in turn, can be inactivated/degraded by other MMPs or members of other classes of proteases, according to a multi-faced regulatory circuit [51,52].

Moreover, the function of active MMPs is controlled by endogenous inhibitors, which include serum globulins and the Tissue Inhibitors of MMPs (TIMPs) [51,52,69]. The latter are present in most human tissues, where they inhibit MMPs with a 1:1 molar stoichiometry, mainly via the binding of their amino-terminal to the MMP catalytic site [69]. To date, four TIMPs have been identified: they share about 40% sequence homology and hinder all the human MMPs, albeit with variable affinity. In particular, TIMP-3 has the broadest inhibitory spectrum [69], while TIMP-1 or TIMP-2 antagonize mostly MMP-9 or MMP-2 activity, respectively [70].

As for soluble MMPs, also the MT-MMPs are synthesized as latent zymogens (proMT-MMPs): inside the cell, the pro-hormone convertase furin converts proMT-MMPs into active MT-MMPs, which are then expressed on the cell surface, where they can complex TIMPs [54]. TIMP capability of inhibiting MT-MMP activity and the fact that in the absence of TIMPs the MT-MMPs quickly undergo autolysis, reduces the number of fully active MT-MMP molecules on the cell membrane [54].

In summary, consistent with its potency and pleiotropic effects, the activity of MMPs is tightly regulated at both the transcriptional and post-transcriptional level. This occurs in physiological conditions; whereas, inflammatory/degenerative diseases or tumors are characterized by an abnormal expression of MMPs and/or their deregulated function [58,71]. 

In particular, cancer cells synthesize MMPs following the activation of oncogenes, the inactivation of onco-suppressor proteins, the stimulation by growth factors or inflammatory mediators, the generation of reactive oxygen species, and/or the presence of hypoxia [72,73,74,75,76]. Since MMPs are produced in tumor tissues also by fibroblasts and infiltrating inflammatory cells [58,71,77,78], MMP expression and/or activity are increased in tumor lesions as compared to neighbor normal tissues [79,80,81,82,83,84,85,86,87,88].

Most notably, MMPs play an important role in tumor progression. Specifically, they digest cell surface molecules mediating cell-to-cell or cell-to-ECM adhesion (e.g., cadherins or integrins), thus allowing tumor cells to detach from adjacent cells and the ECM [58,71]. Then, MMPs degrade basement membranes and the ECM, preparing a route for cancer cell locomotion and tissue invasion [58,71]. In doing so, MMPs allow cancer cells to reach local blood or lymphatic vessels and penetrate their wall, leading to metastases [58,71]. Following their arrest in the lumen of small vessels, cancer cells penetrate again the vessel wall through MMP action and, in case they escape immune surveillance, give rise to secondary tumors [58,71]. Noteworthy, during all these steps, MMPs sustain cancer cells by releasing sequestered, ECM-bound growth factors, activating growth factor receptors, triggering cell survival-promoting signaling pathways and degrading mediators of apoptosis (e.g., FAS ligand) or anti-tumor immunity (e.g., MHC) [58].

MMPs play an important role also in tumor-associated angiogenesis, by breaking down the blood vessel basement membrane and perivascular ECM, mobilizing endothelial cell precursors from bone marrow and recruiting them in the newly forming vessels [89,90,91,92,93,94,95].

It has to be highlighted, however, that in addition to their tumorigenic effects, MMPs can also exert anti-tumor activities. For example, MMP-13 protects against melanoma metastases [96], although its presence is prognostic of poor survival in gastric cancer patients [87]. Moreover, MMP-8 expression is associated with improved survival of tongue cancer patients [97], even though MMP-8 serum levels correlate with colorectal cancer stage and distant metastases [88].

These contrasting activities of MMPs appear to depend on the cellular source of MMP expression, the extracellular environment, the type of tumor and the stage of the disease [98]. In particular, the protective role of some MMPs in specific types and clinical stages of tumor is likely to result from MMP occasional/paradoxical capability of inhibiting tumor angiogenesis via the degradation of angiogenic growth factor receptors, and/or the generation of anti-angiogenesis molecules deriving from the cleavage of ECM components [94,99].

In conclusion, the balance between MMP pro- and anti-tumorigenic effects is critical for the prognosis of the patients. Therefore, evaluating the different MMPs which are expressed in a particular type and/or stage of a specific tumor and patient is of extreme importance. 

Among the various members of the MMP family, MMP-9, MMP-2 or MT1-MMP are especially involved in the development or progression of uterine cervical tumors, as indicated by the finding that their RNA or protein expression and their activity are extremely low or absent in normal uterine cervix and low-grade CIN, are well detectable in high-grade CIN and they are very high in invasive CC [100,101,102,103,104,105,106,107,108,109,110,111,112]. In addition, expression of these MMPs parallels the angiogenic switch occurring during the evolution of high-grade CIN into CC and positively correlates with vessel density in tumor lesions [100,108,109]. Finally, MMP-9, MMP-2 or MT1-MMP levels in patients’ plasma or cervical smears correlate with poor prognosis and higher tumor stage [103,113]. 

Noteworthy, in normal or transformed epithelial cells the E5, E6 or E7 protein of tumorigenic, HR-HPV down-regulate TIMPs and/or up-regulate MMP-2, MMP-9 or MT1-MMP expression or activity, thereby increasing epithelial cell invasiveness (Figure 1) [114,115,116,117,118,119,120]. These phenomena are likely to depend on E5, E6 or E7 capability of triggering the serine/threonine kinase AKT [6,121,122]. In fact, phosphorylated AKT activates transcription factors promoting *mmp* gene expression (Figure 1) [65,66,67,68].

## 3. Effects of HIV-Protease Inhibitors on Pre-Cancer and Cancer Lesions of the Uterine Cervix

Given the major role of MMPs in tumor-associated cellular invasion and angiogenesis, many MMP inhibitors (MMPIs) have been developed in the past years and exploited as anti-cancer interventions.

Among them are natural MMPIs, such as shark cartilage extract or soy isoflavonoid [123,124] and synthetic MMPIs comprising tetracycline derivatives, bisphosphonates and peptides chelating the zinc ion of MMP catalytic site [125,126,127].

Preclinical studies have shown that both synthetic and natural MMPIs are effective against a variety of tumors, included uterine CC [125,128,129,130,131,132,133,134]. However, clinical trials performed with some of the abovementioned MMPIs have failed due to dose-limiting side effects, inefficacy and/or lack of specificity [123,124,135,136,137,138]. In fact, because of the high similarity in the sequence of MMP catalytic domains, first-generation MMPIs have acted against multiple members of the MMP family, hence inhibiting either the therapeutic targets or anti-targets of a specific tumor [136,138]. 

In spite of these discouraging results and considering MMP role in tumor onset or progression, efforts are currently being made in order to design new MMPIs for oncological therapy. In particular, to specifically counteract the MMPs that are produced by a given tumor at a specific stage of development [98], novel strategies for MMP inhibition are exploiting the blockage of secondary substrate-binding sites (exosites), which show remarkable differences among the various MMPs [139,140].

However, inhibition of MMP activity may not be sufficient, as this strategy does not compromise the binding of MMPs to their cellular targets and the consequent signaling [58,71,91,141,142]. Thus, antibody-based inhibitors are being explored [143]. Alternatively, novel MMPIs could antagonize the molecular pathways leading to MMP expression. In this regard, experimental and clinical evidence points to the HIV-protease inhibitors (HIV-PI) as effective MMPIs.

**Figure 2:**
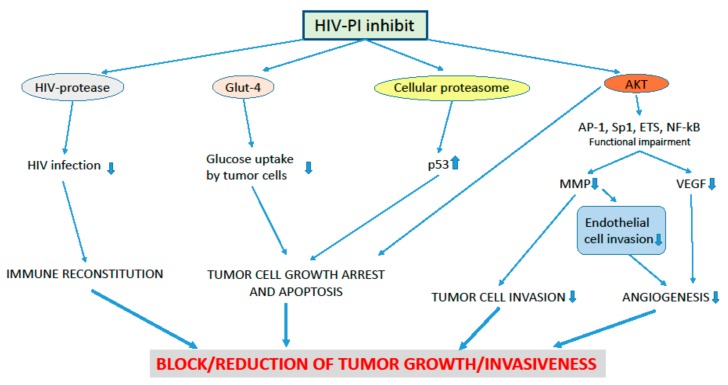
Anti-tumor activities of Human Immunodeficiency Virus (HIV)-protease inhibitors (PI). Arrows symbolize directions of connections. HIV-PI hinder the activity of the: (i) HIV aspartyl protease, hence impeding the production of infectious viral particles and promoting immune reconstitution; (ii) glucose transporter (GLUT)-4, thus impairing glucose uptake by tumor cells; (iii) cellular proteasome, therefore causing p53 protein intracellular accumulation; iv) AKT, this leading to the functional impairment of the Activator Protein (AP)-1, Sp (Specificity protein)-1, ETS or Nuclear Factor-kappa B (NF-κB) transcription factors, the down-regulation of matrix-metalloproteinase (MMP) or vascular endothelial growth factor (VEGF) expression, and the inhibition of angiogenesis or tumor cell invasion. Altogether, these activities of HIV-PI can block or reduce tumor growth and invasiveness.

The HIV-PI are a class of drugs which block the active site of HIV aspartyl protease, hence impeding the cleavage of HIV Gag-Pol poly-protein precursor and, consequently, the production of mature, infectious HIV particles (Figure 2) [144]. 

HIV-PI are administered to HIV-infected people together with the HIV reverse transcriptase inhibitors (HIV-RTI) and/or drugs counteracting the HIV integrase [145]. This combination therapy has been defined as highly active antiretroviral therapy (HAART) [145]. In fact, by potently suppressing viral replication and promoting immune reconstitution, HAART has strongly reduced AIDS-related morbidity and mortality, rendering HIV infection a manageable, chronic disease [146].

Most disappointingly, however, long-term treatment with HIV-PI may cause undesirable side effects. This is because, in addition to the HIV protease, HIV-PI can also target host molecules. Among the off-targets of HIV-PI is the cellular proteasome, a multi-subunit protease complex which controls the turnover of intracellular proteins (Figure 2) [147,148,149,150,151,152]. One of the many consequences resulting from the functional impairment of the cellular proteasome promoted by the HIV-PI is the intracellular accumulation of the sterol regulatory element-binding protein (SREBP) 1 transcription factor [153,154]. As a consequence, SREBP1-targeted lipogenic enzymes are increased, this augmenting lipid plasma levels and altering body fat distribution in treated patients [154,155,156,157]. Another unexpected target of HIV-PI is the glucose transporter (GLUT)-4 (Figure 2), whose inhibition by HIV-PI halts glucose uptake by adipocytes, eventually causing insulin-resistance and diabetes [158]. 

Consequently and considering the need of life-long HAART administration, HIV-PI are often substituted by combined nucleoside and non-nucleoside HIV-RTI, respectively targeting the catalytic and allosteric sites of HIV reverse transcriptase [145].

Either HIV-PI-based or HIV-RTI-based HAART is effective at suppressing HIV infection, rapidly reducing plasma viremia and increasing the number of CD4^+^ T cells in HIV-infected individuals [146]. However, the absence of HIV-PI in HAART deprives patients of the beneficial activities of these drugs which, in addition to the improvement of T cell function [159], comprise the inhibition of AIDS-associated opportunistic infections [160]. 

Moreover, HIV-infected individuals undergoing HAART have experienced a reduced incidence and/or an increased regression of AIDS-associated tumors including Kaposi’s sarcoma (KS) and non-Hodgkin lymphoma (NHL) [161,162,163,164,165]. In contrast, the incidence of progressed uterine CC in HIV-positive women has not decreased since the introduction of HAART [166]. Nevertheless, similarly to KS or NHL, use of HAART in HIV-infected women has decreased the incidence of CIN, caused its regression, and/or lowered its recurrence rates after excision [29,30,31,32,167,168,169,170,171].

The restoration of the immune responses promoted by HAART is likely to have a major role in inhibiting the development of AIDS-associated KS, NHL or CIN (Figure 2) [172]. However, in some HAART-treated, HIV-positive patients the decrease in tumor incidence, relapse or progression rates is not accompanied by the recovery of CD4^+^ T cell number and/or the reduction of viral load [172]. This has suggested that drugs present in HAART could exert anti-tumor effects independently of their capability of suppressing viral replication and reconstituting the immune system.

With reference to uterine CIN or CC, epidemiological studies analyzed all together data collected from patients undergoing different HAART regimens [29,30,31,32,166,167,168,169,170,171]. In contrast, studies concerning AIDS-associated KS have provided better clues of HAART anti-tumor effects. In particular, these studies have reported that complete remission of KS is more frequent in HIV-PI than in HIV-RTI-treated individuals, although both therapeutic regimens can promote the regression of KS lesions [164]. Moreover, a relapse of KS after its resolution has been described in patients switching from HIV-PI-based to HIV-RTI-based HAART [173]. 

In agreement with these findings, results from preclinical (in vitro and in vivo) and clinical studies indicate that HIV-PI can directly inhibit tumor cell survival, growth or invasion and angiogenesis in experimental models devoid of HIV and immune cells.

In particular, due to their inhibitory effect on the function of the cellular proteasome, HIV-PI cause an increase in the intracellular amounts of cell cycle inhibitors or onco-suppressors, hence leading to the growth arrest and/or death of a variety of human tumor cells (Figure 2) [148,150,174,175,176,177,178,179,180,181,182]. Additional anti-cancer cytostatic and/or cytotoxic effects result from HIV-PI capability of inhibiting GLUT-4, hence impairing glucose uptake by tumor cells (Figure 2) [158,183].

As to the uterine cervix, the HIV-PI indinavir (IDV), ritonavir (RTV) or lopinavir have been shown to reduce the viability of HR-HPV-transformed epithelial cells [151]. This is accompanied by a stable increase in the intracellular levels of the proteasome-targeted, growth-arresting and pro-apoptosis p53 protein [151]. However, this activity requires high HIV-PI concentrations, that are not (or only transiently) present in the plasma of treated patients [184].

In the matter of HIV-PI therapeutic (low) amounts, the HIV-PI saquinavir (SQV) or RTV halts the proliferation of cells derived from CIN but not CC lesions, with no significant effect on cell survival or proteasome capability of degrading p53 [185]. In view of these in vitro findings, one may conclude that at low HIV-PI tissue concentrations a CIN lesion persists, although it does not grow. Therefore, in order to efficiently counteract the malignant evolution of high-grade CIN, an HIV-PI should be combined with a classical proteasome inhibitor, as previously done to induce apoptosis of CC-derived cells [186]. Nevertheless, a recent clinical study conducted in HIV-negative women has shown that HIV-PI can by themselves promote the regression or the complete remission of high-grade CIN lesions [187].

Preclinical work has suggested that this may depend on HIV-PI capability of blocking epithelial basement membrane invasion by the CIN cells, as this event starts CIN progression to CC [2,11].

In particular, SQV or RTV at levels as detected in the plasma of treated patients can efficiently inhibit EGF-promoted invasion of primary keratinocytes bearing episomal HR-HPV DNA and derived from low-grade CIN lesions of HIV-negative women [185,188]. For both SQV and RTV, this effect is accompanied by a significant reduction of MMP-2 or MMP-9 activity released by the CIN cells in response to EGF [185,188]. 

These in vitro results are consistent with those from animal studies which have indicated that HIV-PI therapeutic concentrations inhibit the growth of human tumors in animal models via a proteasome-independent reduction of cancer cell invasion and MMP activity (Figure 2) [189].

In agreement with the fact that the HIV protease does not belong to MMP functional class, HIV-PI cannot directly antagonize MMP activity [190]. Rather, SQV or RTV therapeutic levels down-regulate the expression of MMP-9 induced in CIN cells by EGF and, to a lesser extent, CIN cell constitutive expression of MMP-2 [185,188]. This finding is consistent with HIV-PI capability of inhibiting MMP expression by both tumor and normal cells [150,152,189,191,192] and either in HIV-positive or in HIV-negative individuals [193,194].

EGF induces MMP-9 expression by epithelial cells via the phosphorylation of the serine/threonine kinase AKT [195] and the consequent activation of Fos-related antigen (Fra)-1, a member of the AP-1 transcriptional complex [67]. Strikingly, exposure of CIN cells to the same SQV or RTV doses reducing EGF-promoted cellular invasion and MMP-9 expression, also inhibits AKT phosphorylation and Fra-1 nuclear localization triggered in these cells by EGF [188]. These findings are consistent with results obtained in other experimental models, which indicate that inhibition of EGF-induced AKT phosphorylation, or Fra-1 silencing, reduces cellular invasion via the down-regulation of MMP-9 expression [67,195].

Indeed, SQV or RTV capability of inhibiting EGF-promoted AKT/Fra-1 activation and MMP-9 expression in low-grade CIN cells may have relevant clinical implications. In fact, the levels of EGF, MMP-9 and phosphorylated AKT are extremely low or undetectable in the normal uterine cervix, becoming appreciable in low-grade CIN and further increasing during its evolution to high-grade CIN and CC [103,107,113,196,197,198].

## 4. The Anti-Angiogenesis Effect of the HIV-PI and its Possible Impact on Pre-Cancer and Cancer Lesions of the Uterine Cervix

As for the CIN cells, HIV-PI can impair AKT phosphorylation in several tumor cell types (Figure 2) [150,182,199,200,201,202,203,204]. In this regard, it has to highlighted that the activation of AKT is pivotal in the acquisition and maintenance of cancer hallmarks, including cellular immortalization, deregulated growth and invasiveness, as well as in angiogenesis [65,67,68,205,206,207]. 

Because of its role in the development and clinical progression of uterine cervical lesions [22,23,100], angiogenesis is a rational target for the treatment of high-grade CIN and/or CC. Accordingly, antagonists of angiogenic growth factors have been found active against recurrent CC [20]. However, these antagonists are very expensive [20] and their therapeutic use may lead to drug resistance [208]. This has prompted the exploitation of drugs counteracting other players of the angiogenic process.

In this context, the HIV-PI nelfinavir reduces the production of the angiogenic VEGF (Figure 2) [179,204,209]. Moreover, SQV or IDV blocks MMP activity in endothelial cells, hence halting the first step of angiogenesis that is endothelial cell invasion of vessel basement membrane (Figure 2) [191]. As a consequence, nelfinavir, SQV or IDV efficiently counteract the formation of new blood vessels in HIV-free in vivo models [189,191,209,210]. Consistently, IDV can promote the regression or stabilization of proliferative vascular lesions also in HIV-negative KS patients [194]. 

In vitro work has shown that IDV concentrations as found in plasma of treated HIV-positive or negative patients inhibit the conversion of latent MMP-2 zymogen into the active enzyme, while having little or no impact on the synthesis of latent MMP-2 and no direct effects against MMP-2 catalytic activity [190,191].

The main mediator of latent MMP-2 conversion into active MMP-2 is the MT1-MMP [54]. Noteworthy, in vitro work has shown that IDV therapeutic amounts impair the binding of the Sp1 transcription factor to the promoter region of the *mt1-mmp* gene, thus down-regulating MT1-MMP RNA and protein expression by endothelial cells [210]. 

It has to be noted that MT1-MMP can directly degrade the blood vessel basement membrane even in the absence of MMP-2 [211]. Moreover, by modulating the expression of endothelial cell adhesion molecules, MT1-MMP is important to endothelial cell organization in a capillary network [211]. Thus, the reduction of MT1-MMP expression provides a molecular mechanism for IDV anti-angiogenesis effect, rendering this anti-HIV drug a promising antagonist of the formation of new blood vessels which accompanies CIN evolution into CC. Notwithstanding, the finding that long term-treatment with HIV-PI can damage blood vessels [212] deserves careful consideration.

## 5. HIV-PI and HPV

As discussed above, the E6 and E7 proteins of HR-HPV exert tumorigenic and pro-angiogenesis activities, including the inactivation of the growth-suppressive pRb or p53 proteins, the phosphorylation of AKT and the promotion of MMP expression (Figure 1) [8,9,114,115,116,118,119,120,121,122]. These activities of HR-HPV-E6 or E7, in turn, lead to cellular growth and invasiveness (Figure 1). Nevertheless, the anti-proliferative and anti-invasive effects exerted in CIN cells by SQV or RTV therapeutic amounts are not accompanied by the down-regulation of HPV-E6 or E7 expression [188]. This is consistent with the clinical finding that HIV-PI promote CIN regression without clearing HPV infection [167], albeit other studies have obtained opposite results [32,187].

In this regard, in vitro work indicates that therapeutic concentrations of HIV-PI are active against CIN cells containing episomal HPV DNA, while having little or no effect on CC cells bearing integrated HPV-DNA [185,188].

Therefore, one may speculate that the activity of HIV-PI against HPV-associated cervical lesions could depend on the integration status of HPV-DNA. This hypothesis is corroborated by molecular studies indicating that HIV-PI selectively accumulate in the nuclei of HPV-E6 expressing cells and trigger the synthesis of anti-viral proteins [213,214]. 

Thus, HIV-PI could counteract the early stage of HPV infection, when cervical epithelial cells are not heavily transformed, HPV-DNA is episomic and the expression of E6, E7 and other HPV proteins is low. In this specific setting, HIV-PI could also favor the clearance of episomal HPV DNA. This suggests that HIV-PI should be used early in the clinical management of CIN and, possibly, prior to transition to CC.

## 6. Concluding Remarks and Future Directions

HPV-associated uterine CC is a serious health problem which can be prevented firstly by anti-HPV vaccination [215] and, secondly, through screening programs for the detection and surveillance of early lesions and the removal of high-grade CIN [216].

These prevention programs are feasible and largely applied in the developed but not in the developing world [217]. Consequently, low-income countries are now coping with a burden of uterine CC, especially in association with HIV infection [217].

With reference to CIN therapeutic approaches, however, it has to be noted that surgical removal of high-grade lesions may lead to uterine ulcers, hemorrhages, cicatrices and pre-term labor [218]. In this context, results from preclinical and clinical studies point to HIV-PI as a promising intervention in the management of high-grade CIN lesions of both HIV-infected and uninfected women. In fact, HIV-PI directly inhibit events leading to CIN onset and progression including the growth or invasion of HPV-positive epithelial cells [151,185,188] and angiogenesis [189,191,210]. These effects are independent of the anti-HIV and immune-reconstituting activities of HIV-PI. Consistently, as for HIV-positive patients, HIV-PI causes the regression or the complete remission of high-grade CIN lesions also in HIV-negative women [187].

Indeed, HIV-PI counteract key players of carcinogenesis, such as the cellular proteasome, the serine/threonine kinase AKT and the MMPs (Figure 2) [147,148,149,150,151,152,182,185,188,189,190,191,192,199,200,201,202,203,204,210]. As the cellular proteasome has a role in regulating AKT phosphorylation and MMP expression [152,219,220], a connection among the pathways targeted by the HIV-PI may exist.

At the present time, novel and tailor-made inhibitors of the cellular proteasome, AKT or MMPs are under evaluation [139,140,143,221,222,223], since the first generation of these drugs has given unsatisfactory results [123,124,135,136,137,138,224,225].

In this regard, however, it should be considered that differently from novel proteasome, AKT or MMP inhibitors, HIV-PI are in use since many years and information on their pharmacokinetic, tissue distribution and toxicity profile is large [184,226]. 

Regarding the use of HIV-PI in oncology, this should entail tumor-customized therapies. In fact, each member of the HIV-PI class of drugs seems to have distinct effects on MMPs, while each tumor produces different MMPs, depending on its type and stage of progression. 

As to uterine CIN, MMP-9, MT1-MMP and MMP-2 have a key role in its evolution into CC [100,101,102,103,104,105,106,107,108,109,110,111,112,113]. Noteworthy, either SQV or RTV down-regulates MMP-9 expression, thereby blocking CIN cell invasiveness [185,188]; whereas, IDV reduces MT1-MMP synthesis, thus impairing MMP-2 functional activation and angiogenesis [191,210].

Therefore, in order to promote the regression of high-grade CIN, administrating a combination of different HIV-PI may be advisable and effective, as indicated by a recent clinical study [187]. 

Although the systemic use of HIV-PI has been reported to cause the regression or the complete remission of KS or CIN in the absence of adverse side effects [187,194], topical application of these drugs may be safer and effective, against high-grade CIN. This therapeutic strategy can be applicable worldwide.

Future work will identify HIV-PI chemical groups responsible for their anti-tumor effects, hence leading to the synthesis of more active, less toxic derivatives.

In this context, it has to be highlighted that HIV-PI inhibitory effect on MMPs impairs events which are key for high-grade CIN evolution into CC, namely CIN and endothelial cell invasion of the basement membranes. Therefore, labeled probes utilizing MMP substrate specificity for imaging purposes [227,228,229] could allow the monitoring of the therapeutic response to HIV-PI or their future derivatives.
